# Correlation between *PPARG* Pro12Ala Polymorphism and Therapeutic Responses to Thiazolidinediones in Patients with Type 2 Diabetes: A Meta-Analysis

**DOI:** 10.3390/pharmaceutics15061778

**Published:** 2023-06-20

**Authors:** Eun Jeong Jang, Da Hoon Lee, Sae-Seul Im, Jeong Yee, Hye Sun Gwak

**Affiliations:** 1 College of Pharmacy and Graduate School of Pharmaceutical Sciences, Ewha Womans University, 52 Ewhayeodae-gil, Seodaemun-gu, Seoul 03760, Republic of Korea; heimdall01@hanmail.net (E.J.J.); hhooonnn@ewhain.net (D.H.L.); jjjhello1@naver.com (J.Y.); 2 Graduate School of Clinical Biohealth, Ewha Womans University, Seoul 03760, Republic of Korea; cecillm0526@gmail.com

**Keywords:** polymorphism, PPARG, thiazolidinedione, type 2 diabetes

## Abstract

**Background:** Thiazolidinediones (TZDs) are a type of oral drug that are utilized for the treatment of type 2 diabetes mellitus (T2DM). They function by acting as agonists for a nuclear transcription factor known as peroxisome proliferator-activated receptor-gamma (PPAR-γ). TZDs, such as pioglitazone and rosiglitazone, help enhance the regulation of metabolism in individuals with T2DM by improving their sensitivity to insulin. Previous studies have suggested a relationship between the therapeutic efficacy of TZDs and the *PPARG* Pro12Ala polymorphism (C > G, rs1801282). However, the small sample sizes of these studies may limit their applicability in clinical settings. To address this limitation, we conducted a meta-analysis assessing the influence of the *PPARG* Pro12Ala polymorphism on the responsiveness of TZDs. **Method:** We registered our study protocol with PROSPERO, number CRD42022354577. We conducted a comprehensive search of the PubMed, Web of Science, and Embase databases, including studies published up to August 2022. We examined studies investigating the association between the *PPARG* Pro12Ala polymorphism and metabolic parameters such as hemoglobin A_1C_ (HbA_1C_), fasting plasma glucose (FPG), triglyceride (TG), low-density lipoprotein cholesterol (LDL), high-density lipoprotein cholesterol (HDL), and total cholesterol (TC). The mean difference (MD) and 95% confidence intervals (CIs) between pre- and post-drug administration were evaluated. The quality of the studies included in the meta-analysis was assessed by using the Newcastle–Ottawa Scale (NOS) tool for cohort studies. Heterogeneity across studies was assessed by using the *I*^2^ value. An *I*^2^ value greater than 50% indicated substantial heterogeneity, and a random-effects model was used for meta-analysis. If the *I*^2^ value was below 50%, a fixed-effects model was employed instead. Both Begg’s rank correlation test and Egger’s regression test were performed to detect publication bias, using R Studio software. **Results:** Our meta-analysis incorporated 6 studies with 777 patients for blood glucose levels and 5 studies with 747 patients for lipid levels. The included studies were published between 2003 and 2016, with the majority involving Asian populations. Five of the six studies utilized pioglitazone, while the remaining study employed rosiglitazone. The quality scores, as assessed with the NOS, ranged from 8 to 9. Patients carrying the G allele exhibited a significantly greater reduction in HbA_1C_ (MD = −0.3; 95% CI = −0.55 to −0.05; *p =* 0.02) and FPG (MD = −10.91; 95% CI = −19.82 to −2.01; *p =* 0.02) levels compared to those with the CC genotype. Furthermore, individuals with the G allele experienced a significantly larger decrease in TG levels than those with the CC genotype (MD = −26.88; 95% CI = −41.30 to −12.46; *p =* 0.0003). No statistically significant differences were observed in LDL (MD = 6.69; 95% CI = −0.90 to 14.29; *p =* 0.08), HDL (MD = 0.31; 95% CI = −1.62 to 2.23; *p =* 0.75), and TC (MD = 6.4; 95% CI = −0.05 to 12.84; *p =* 0.05) levels. No evidence of publication bias was detected based on Begg’s test and Egger’s test results. **Conclusions:** This meta-analysis reveals that patients with the Ala12 variant in the *PPARG* Pro12Ala polymorphism are more likely to exhibit positive responses to TZD treatment in terms of HbA_1C_, FPG, and TG levels compared to those with the Pro12/Pro12 genotype. These findings suggest that genotyping the *PPARG* Pro12Ala in diabetic patients may be advantageous for devising personalized treatment strategies, particularly for identifying individuals who are likely to respond favorably to TZDs.

## 1. Introduction

Type 2 diabetes mellitus (T2DM) is a widespread metabolic disorder characterized by beta-cell dysfunction and insulin resistance, significantly impacting patients’ quality of life. T2DM currently affects over 400 million individuals worldwide, with projections estimating an increase to 552 million cases by 2030 [1]. The primary pharmacological treatment for T2DM consists of medications such as sulfonylureas, biguanides, γ-glucosidase inhibitors, sodium glucose co-transporter-2 inhibitors, and thiazolidinediones (TZDs). In cases in which patients are unable to achieve the desired therapeutic outcomes with a single oral hypoglycemic agent, combination therapy involving two drugs is often recommended [2].

Thiazolidinediones (TZDs) are oral medications that are used to treat T2DM by acting as agonists for the nuclear transcription factor called peroxisome proliferator-activated receptor-gamma (PPAR-γ). TZDs, including pioglitazone and rosiglitazone, enhance metabolic regulation in patients with T2DM by improving insulin sensitivity [3,4]. Early research on T2DM suggested that TZDs lower hyperglycemia and hyperinsulinemia levels while increasing insulin sensitivity in the liver, adipose tissue, and skeletal muscle [5,6]. TZDs may also have a beneficial effect on preserving beta-cell function or mass, which is responsible for insulin production, compared to insulin secretagogues such as sulfonylureas [7]. Additionally, TZDs have been found to reduce the urinary excretion of albumin and other proteins and hinder the development and advancement of diabetic nephropathy [8].

Moreover, TZDs possess significant effects beyond glycemic control, including their influence on lipid metabolism modulation. TZDs decrease free fatty acid (FFA) levels and significantly reduce triglyceride concentrations due to the critical role of PPAR-γ in regulating adipocyte gene expression, which is associated with obesity [9]. These effects have been observed in animal models with hyperinsulinemia and in non-diabetic animals, indicating that the mechanism operates independently of glucose-lowering action [10]. Debril et al. [11] demonstrated that PPAR-γ influences lipid homeostasis by regulating the gene transcription that is involved in lipid metabolism and storage. Considering that dyslipidemia plays a crucial role as a risk factor for atherosclerosis, the therapeutic modulation of lipid levels may protect against cardiovascular disease in patients with T2DM.

In clinical practice, substantial fluctuations in hemoglobin A_1C_ (HbA_1C_, %) and fasting plasma glucose (FPG, mg/dL) levels are frequently observed in patients treated with TZDs [12,13]. Although TZDs provide notable therapeutic advantages, the conventional dosage forms of TZDs yield inconsistent efficacy among patients, potentially resulting in increased adverse effects. This variability in response can contribute to the inefficacy of the treatment and non-compliance among patients. Individual differences in glycemic control and treatment response may result from various factors, including genetic factors [14].

The *PPARG* gene encodes the PPAR-γ protein, a transcription factor that regulates the genes that are essential for maintaining lipid and glucose homeostasis [11]. The activation of PPAR-γ in adipocytes promotes the proper and balanced secretion of adipocytokines, such as adiponectin and leptin, which help mediate insulin action in peripheral tissues and maintain insulin sensitivity [15]. The *PPARG* Pro12Ala polymorphism (rs1801282, C > G) is a well-known genetic variation, with this missense variant observed to affect the transcriptional activity of PPAR-γ [16]. A prior meta-analysis showed significant associations between the *PPARG* Pro12Ala polymorphism and T2DM risk, reporting that the Ala12 polymorphism was associated with a reduced risk of T2DM [17].

The *PPARG* Pro12Ala polymorphism may influence the treatment efficacy of TZDs, which serve as ligands for PPAR-γ. Given the lack of meta-analyses on the polymorphism’s impact on changes in blood glucose and lipid profiles, this study aims to meta-analyze the results of the relationship between the *PPARG* Pro12Ala polymorphism and changes in HbA_1C_, FPG, triglyceride (TG, mg/dL), low-density lipoprotein cholesterol (LDL, mg/dL), high-density lipoprotein cholesterol (HDL, mg/dL), and total cholesterol (TC, mg/dL) from baseline through this meta-analysis.

## 2. Materials and Methods

### 2.1. Protocol and Registration

The study protocol was registered with PROSPERO under the number CRD42022354577.

### 2.2. Search Strategy and Study Selection

This study adhered to the guidelines outlined by the Preferred Reporting Items for Systematic Reviews and Meta-Analyses (PRISMA) [18]. A systematic search was conducted independently by two investigators across three electronic databases (Web of Science, PubMed, and Embase) from 1 September 2022 to 29 November 2022. The search utilized the following keywords: (thiazolidinedion* OR (thiazolidine dion*) OR TZD OR glitazon* OR pioglitazon* OR rosiglitazon* OR troglitazon* OR lobeglitazon*) AND (polymorph* OR variant* OR mutation* OR genotyp* OR haplotyp* OR allele* OR SNP* OR pharmacogen* OR Pro12Ala OR rs1801282) AND ((peroxisome proliferator activated receptor gamma) OR (peroxisome proliferator activated receptor γ) OR (PPAR gamma) OR (PPARgamma) OR (PPARγ) OR (PPAR γ) OR PPARG) AND ((glycemic OR glycaemic OR glucos*) OR (lipid* OR cholesterol* OR lipoprotein*) OR ((glycated hemoglobin) OR (glycated haemoglobin) OR (glycosylated hemoglobin) OR (glycosylated haemoglobin) OR (hemoglobin A1c) OR (haemoglobin A1c) OR HbA1c OR A1c OR glycohemoglobin OR glycohaemoglobin) OR ((fasting plasma glucos*) OR FPG OR (fasting blood glucos*) OR FBG OR (fasting blood sugar)) OR ((low density lipoprotein cholesterol*) OR (LDL cholesterol*) OR (LDL C) OR (low density lipoprotein*) OR LDL OR (beta lipoprotein cholesterol*)) OR ((high density lipoprotein cholesterol*) OR (HDL cholesterol*) OR (HDL C) OR (high density lipoprotein*) OR HDL OR (alpha lipoprotein cholesterol*)) OR (triglycerid* OR triacylglycerol* OR TG) OR ((total cholesterol*) OR TC)) (Supplementary Table S1).

Inclusion criteria for the study selection were as follows: (1) studies providing sufficient information on the impact of *PPARG* Pro12Ala polymorphism on responses to TZDs in T2DM patients; (2) studies including any of HbA_1C_, FPG, TC, HDL, LDL, or TG levels as outcomes; and (3) articles published in English.

Following the removal of duplicates, the titles and abstracts of the remaining studies were independently reviewed by two researchers to exclude irrelevant studies. Subsequently, the full-text articles were assessed for eligibility. Disagreements between the two researchers were resolved through consensus.

### 2.3. Data Extraction and Quality Assessment

Two independent researchers extracted data from the selected studies by using a predetermined spreadsheet for data recording. The extracted data comprised the following information: first author’s name, publication year, ethnicity, patient count, male ratio, mean age, mean baseline BMI, types and dosages of TZDs, time to outcome extraction, genotyping method, and outcomes measured. Additionally, two reviewers independently evaluated the quality of the studies included in the meta-analysis by using the Newcastle–Ottawa Scale (NOS) tool for cohort studies [19]. The NOS tool assesses quality based on three categories: subject selection (up to 4 points), case and control group comparability (up to 2 points), and outcome assessment (up to 3 points). Each item in the categories is assigned a score ranging from one point, except for comparability, which can be adjusted based on the specific topic under investigation, allowing for a maximum score of two points.

### 2.4. Statistical Analysis

Mean differences (MD) and 95% confidence intervals (CIs) were utilized to evaluate the impact of the *PPARG* Pro12Ala polymorphism on each of the six outcomes (HbA_1C_, FPG, TC, HDL, LDL, and TG) of TZDs, with the *Z*-test performed to ascertain the statistical significance of the results. To obtain pooled estimates, the mean and standard deviation were extracted. The Cochrane Handbook [20] presented the formula for calculating the mean and standard deviation of changes. The formulas that were developed by Higgins et al. [21] were applied to convert log-transformed mean and standard deviation data into the raw scale. Heterogeneity across studies was assessed by using the *I*^2^ value [22]. An *I*^2^ value greater than 50% indicated substantial heterogeneity, and a random-effects model was used for meta-analysis. If the *I*^2^ value was below 50%, a fixed-effects model was employed instead. Sensitivity analysis was conducted by excluding each study to evaluate the impact of individual studies on the overall result. Both Begg’s rank correlation test and Egger’s regression test were performed to detect publication bias by using R Studio software (version 4.3.0; R Foundation for Statistical Computing, Vienna, Austria) [23,24]. The meta-analysis was executed by using Review Manager (RevMan), version 5.4 (The Cochrane Collaboration, Copenhagen, Denmark). Statistical significance was defined by a *p*-value < 0.05.

## 3. Results

**Figure 1:**
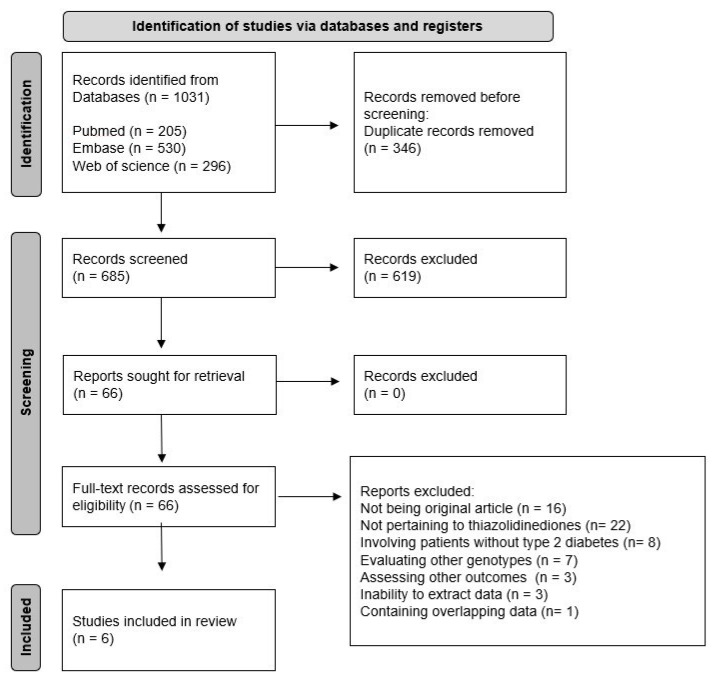
Flow diagram illustrating the study selection process.

A total of 1031 studies was identified in the initial search. After the removal of 346 duplicates, 685 records underwent screening based on titles and abstracts. Of these, 619 studies were excluded, leaving 66 studies for full-text review. Subsequently, 60 studies were excluded for reasons such as not being an original article (n = 16), not pertaining to TZDs (n = 22), involving patients without T2DM (n = 8), evaluating other genotypes (n = 7), assessing other outcomes (n = 3), inability to extract data (n = 3), or containing overlapping data (n = 1). Consequently, 6 studies [25,26,27,28,29,30] involving 777 patients were included in the meta-analysis. The study selection process is illustrated in Figure 1, and the characteristics of the included studies are summarized in Table 1.

The included studies were published between 2003 and 2016, with the majority involving Asian populations. Five of the six studies utilized pioglitazone, while the remaining study employed rosiglitazone. The studies were predominantly conducted on patients aged 50 and above, with a range of an average age between 51.44 and 60.7. Of the six studies, two excluded patients with current exposure to other antidiabetic medication or who required concomitant therapy with other antidiabetic drugs [25,29]. One excluded patients who were receiving insulin treatment [27]. The quality scores, as assessed with the NOS, ranged from 8 to 9. Although other studies provided data on both glucose and lipid levels, Priya et al. [30] reported data on glucose levels only.

**Figure 2:**
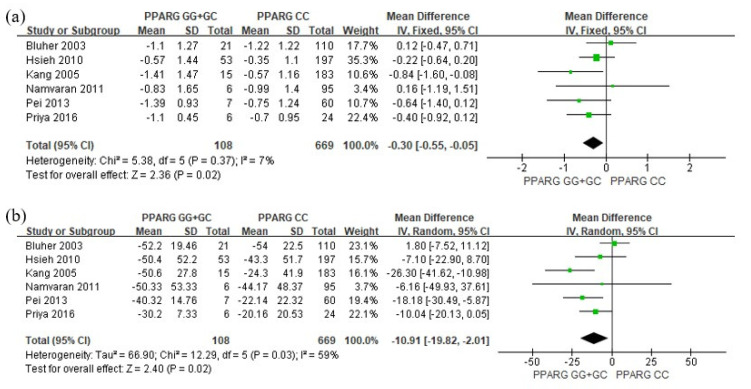
Forest plots showing the association between *PPARG* rs1801282 and changes in (**a**) HbA_1C_ and (**b**) FPG levels from baseline [25,26,27,28,29,30].

**Figure 3:**
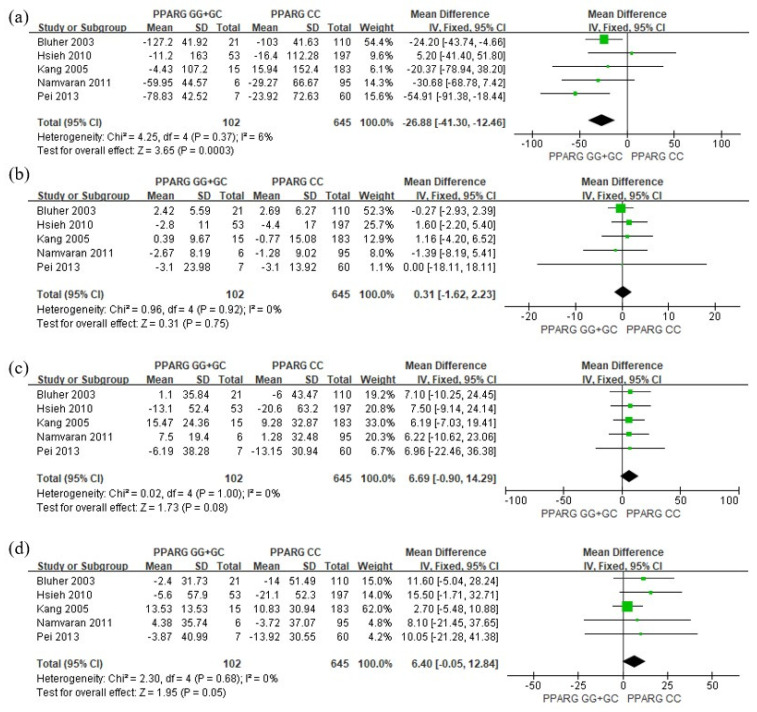
Forest plots showing the association between *PPARG* rs1801282 and changes in (**a**) TG, (**b**) HDL, (**c**) LDL, and (**d**) TC levels from baseline [25,26,27,28,29,30].

**Figure 4:**
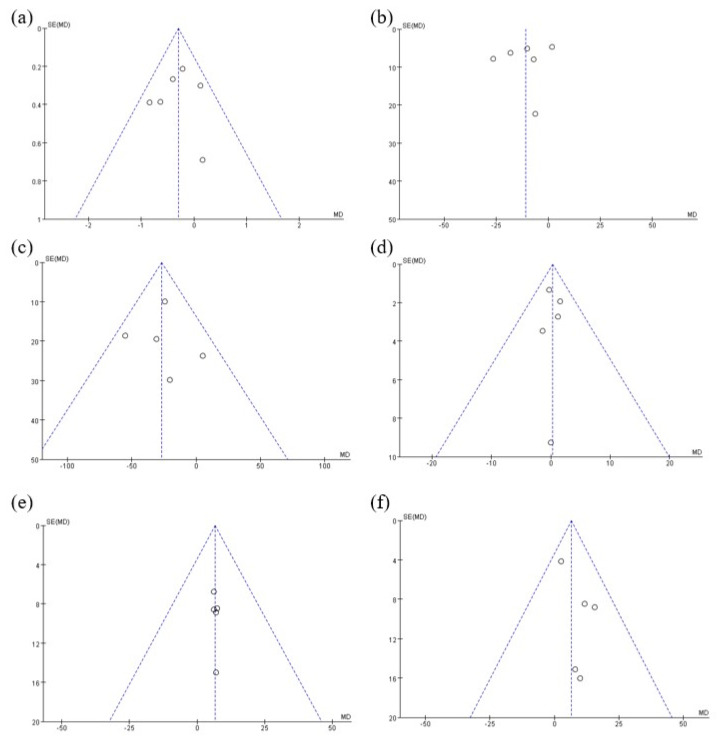
Funnel plots showing the association between *PPARG* rs1801282 and changes in (**a**) HbA_1C_, (**b**) FPG, (**c**) TG, (**d**) HDL, (**e**) LDL, and (**f**) TC levels from baseline.

The meta-analysis results examining the effect of *PPARG* Pro12Ala polymorphism (C > G, rs1801282) on blood glucose and lipid concentrations are depicted in Figure 2 and Figure 3, respectively. With regard to glucose levels, the G allele carriers exhibited a significant reduction in HbA_1C_ (MD = −0.3; 95% CI = −0.55 to −0.05; *p =* 0.02; *I*^2^ = 7%, Figure 2a) and FPG (MD = −10.91; 95% CI = −19.82 to −2.01; *p =* 0.02; *I*^2^ = 59%, Figure 2b) compared to patients carrying the CC genotype. For lipid levels, G allele carriers demonstrated a significant decrease in TG relative to CC genotype patients (MD = −26.88; 95% CI = −41.30 to −12.46, *p =* 0.00003, *I*^2^ = 6%, Figure 3a). Although there was no statistically significant difference in HDL (MD = 0.31; 95% CI = −1.62 to 2.23; *p =* 0.75; *I*^2^ = 0%, Figure 3b) and LDL (MD = 6.69; 95% CI = −0.90 to 14.29; *p =* 0.08; *I*^2^ = 0%, Figure 3c), TC (MD = 6.4; 95% CI = −0.05 to 12.84; *p =* 0.05; *I*^2^ = 0%, Figure 3d) exhibited a marginally significant increase in G allele carriers. The funnel plots were asymmetrical (Figure 4), and no evidence of publication bias was detected based on Begg’s test and Egger’s test results (Supplementary Table S2).

Sensitivity analyses were conducted to evaluate the impact of each study on the pooled estimate. These analyses involved excluding individual studies one by one and then recalculating the pooled MD estimates based on the remaining studies. This approach allowed for an assessment of how each study influenced the combined estimate, providing insights into the robustness and reliability of the results. Excluding the study by Bluher et al. [25] led to a reduction in *I*^2^ values for the FPG result from 59% (*p =* 0.02) to 8% (*p <* 0.0001) (Supplementary Table S3). Except for the study by Kang et al. [27], the TC outcome reached statistical significance (MD = 12.43; 95% CI = 1.97 to 22.88; *p =* 0.02; *I*^2^ = 0%, Supplementary Table S4). Sensitivity analysis did not reveal any substantial change in statistical significance for the results of HbA_1C_, TG, HDL, and LDL (Supplementary Tables S5–S8).

## 4. Discussion

This meta-analysis investigated the effects of the *PPARG* Pro12Ala polymorphism on the response to TZDs in patients with T2DM. Patients carrying the Ala12 allele experienced a more significant reduction in HbA_1C_, FPG, and TG levels by 0.3%, 10.91 mg/dL, and 26.88 mg/dL, respectively. However, the results for HDL, LDL, and TC did not show a significant difference.

TZDs function as insulin sensitizers, enhancing glucose uptake in insulin-insensitive tissues. These effects are achieved primarily through their selective binding to PPAR-γ receptors in adipose tissue [31]. Activation of PPAR-γ by TZDs has been shown to decrease circulating FFA levels by inducing adipocyte differentiation and apoptosis and upregulating the enzyme responsible for FFA transport into adipocytes [32]. This activation reduces lipolysis and lowers circulating FFA levels, resulting in an expansion of adipose tissue mass [33]. The increase in adipose tissue mass serves as a protective mechanism for insulin-sensitive tissues, including the liver, skeletal muscle, and, potentially, pancreatic beta cells, shielding them from insulin resistance [2]. Consequently, glucose metabolism in the liver and muscle improves. Furthermore, the detrimental effects of elevated fat content on pancreatic beta cells are diminished, resulting in reduced beta-cell apoptosis, improved beta-cell mass, and enhanced insulin secretion in individuals with T2DM [7].

Numerous clinical studies have established that TZDs effectively reduce HbA_1C_ and FPG levels as monotherapy or in combination with other antidiabetic drugs compared to placebo groups. Filipova et al.’s meta-analysis [34] reported statistically significant reductions in FPG and HbA_1C_ levels of approximately 18.8 to 36 mg/dL and −0.9 to −1.3%, respectively, in patients treated with pioglitazone (*p <* 0.0001 for both outcomes). Additionally, rosiglitazone 4 mg twice daily as monotherapy significantly reduced HbA_1C_ by 1.5% and FPG by 76 mg/dL compared with the placebo group (*p <* 0.0001, both) [35].

PPAR-γ agonists also have the ability to regulate several genes including adiponectin, tumor necrosis factor-alpha, resistin, and 11β-hydroxysteroid dehydrogenase 1. Adiponectin, an adipocytokine that is produced primarily by adipose tissue, is found to have reduced levels in individuals with obesity, T2DM, and lipodystrophy [36]. Several studies have established a correlation between adiponectin and plasma lipid levels [37,38,39,40]. In the majority of these studies, the levels of adiponectin have demonstrated an inverse relationship with LDL, TG, and TC, while exhibiting a positive correlation with HDL. Additionally, it has been observed that plasma adiponectin plays a regulatory role in the metabolism of TG-rich lipoproteins and lipid metabolism regulatory enzymes [41]. TG and TG-rich lipoproteins increase the risk of myocardial infarction, ischemic stroke, and aortic valve stenosis. These data indicate that TG may contribute significantly to residual cardiovascular risk in patients with T2DM [40]. TZDs modulate adiponectin production, which has been shown to increase lipoprotein lipase synthesis and promote fatty acid oxidation, thus reducing TG concentration [42]. A study found that both rosiglitazone (*p =* 0.026) and pioglitazone (*p =* 0.004) groups with T2DM exhibited a significant increase in adiponectin levels compared to the placebo group, resulting in decreased TG concentration [43].

Previous research has reported that a *PPARG* Pro12Ala polymorphism was associated with dyslipidemia and cardiovascular disease [44] because PPAR-γ regulates the expression of key proteins involved in lipid metabolism. This research demonstrated that the presence of the Ala allele was linked to genetic susceptibility to hypertension in an Asian population. There was a significant difference in the frequency of the Ala allele between individuals with hypertension with elevated blood lipids and the control group (*p =* 0.04). These findings suggest a potential association between the *PPARG* Pro12Ala polymorphism and an increased risk of dyslipidemia. In addition, Li et al. [45] aimed to assess the association between the *PPARG* Pro12Ala polymorphism and serum lipid levels in a meta-analysis. The study demonstrated that individuals carrying the Ala12 allele had TG levels that were consistently lower by 5.31 mg/dL compared to those with the Pro12/Pro12 genotype, regardless of whether they were from Asian or non-Asian populations, while TC and LDL levels increased significantly by 0.77 mg/dL each in the Asian population. These results suggest a potential relationship between the *PPARG* Pro12Ala polymorphism and lipid profiles, particularly TG, TC, and LDL levels.

An in vitro study suggested that the *PPARG* Pro12Ala polymorphism resulted in reduced PPAR-γ activation and adipogenesis compared to the wild type [16]. Given that TZDs act by activating PPAR-γ, it was expected that Ala12 carriers would have lower effects than Pro12/Pro12 carriers. However, our meta-analysis found the opposite. Yamauchi et al. [46] uncovered mechanisms by which both PPAR-γ agonists and PPAR-γ deficiency improved insulin resistance. PPAR-γ activation through TZDs led to a decrease in TG content in the liver and muscle while increasing white adipose tissue. Furthermore, TZDs stimulated adipocyte differentiation and apoptosis, resulting in a decrease in insulin-resistance-inducing substances. Conversely, reduced PPAR-γ activity through *PPARG* deficiency could decrease TG content in white adipose tissue, skeletal muscle, and liver by increasing leptin expression. This may explain why T2DM patients with the PPAR-γ Pro12Ala polymorphism are associated with a decrease in HbA_1C_, FPG, and TG when taking TZDs.

In the sensitivity analysis, heterogeneity in the FPG analysis was diminished upon exclusion of the Bluher et al. study [25]. This reduction may be attributed to the fact that this study focused on European populations, whereas the other studies targeted Asian populations. Additionally, unlike other studies, Bluher et al.’s study involved participants with obesity (mean BMI > 30), potentially contributing to the heterogeneity. Regarding TC, statistical significance was attained when the Kang et al. study [27] was omitted. This result may be due to the exclusive use of rosiglitazone treatment in this study, while the other investigations employed pioglitazone treatment. Further research is necessary to determine if there are differences between pioglitazone and rosiglitazone in their effects on lipid profiles according to the *PPARG* Pro12Ala genotype.

Recently, Vallo et al. [47] conducted a meta-analysis of four studies to explore the association between the *PPARG* Pro12Ala polymorphism and response to TZD treatment in T2DM patients, utilizing FPG and HbA_1C_ as outcomes. This analysis compared the results only after TZD treatment according to the *PPARG* Pro12Ala polymorphism, without examining the changes in values before and after TZD treatment. The difference values before and after TZD treatment were analyzed only within the Pro12/Pro12 genotype group or within the Ala12 carriers. In contrast, our meta-analysis assessed the changes in HbA_1C,_ FPG, TG, HDL, LDL, and TC levels based on the *PPARG* Pro12Ala polymorphism.

This meta-analysis presents several limitations. First, since only six studies were included, the statistical power and external validity of the study may be low. Therefore, caution should be exercised when applying these findings to other patient populations. Second, there is a lack of relatively recent investigations. Third, unpredictable errors may have occurred while calculating the differences between pre- and post-results to determine changes from the baseline. Fourth, due to the lack of information from individual studies, some potential factors which could affect the patient’s TZD response (e.g., patient’s lifestyle or comorbidities) could not be adjusted. Lastly, a meta-analysis of the TC:HDL ratio as an outcome was not possible due to insufficient data [48]. Nevertheless, this is the first systematic review and meta-analysis to evaluate the effects of *PPARG* Pro12Ala polymorphism on changes in blood glucose and lipid. This study can serve as a basis for providing personalized drug therapy to individual patients by identifying patients who may respond better to glucose and lipid level control among those receiving TZD treatment through *PPARG* Pro12Ala genotyping.

## 5. Conclusions

In this meta-analysis, we discovered that the *PPARG* Pro12Ala polymorphisms exerted a statistically significant impact on TZD-induced changes in HbA_1C_, FPG, and TG levels in T2DM patients. Consequently, PPAR-γ genotyping may assist in optimizing T2DM management of blood glucose and lipids for individual patients, ultimately leading to improved treatment outcomes.

## Figures and Tables

**Table 1:** Characteristics of studies included.

First Author, Year	Ethnicity	N (Male %)	Age, Year (SD)	Baseline BMI (SD)	TZD Treatment, mg/Day	Time to Outcome Extraction (Weeks)	Genotyping Methods	Outcomes Measured	Total NOS
Bluher et al., 2003 [25]	European	131 (54.2)	60.7 (9.3)	31.0 (3.3)	Pioglitazone, 45	26	PCR	HbA_1C_, FPG, TG, HDL, LDL, TC	9
Hsieh et al., 2010 [26]	Asian	250 (47.6)	57.86 (11.5)	26.52 (4.4)	Pioglitazone, 30	24	PCR-RFLP	HbA_1C_, FPG, TG, HDL, LDL, TC	8
Kang et al., 2005 [27]	Asian	198 (52.5)	56.5 (9.3)	26.1 (2.8)	Rosiglitazone, 4	12	PCR	HbA_1C_, FPG, TG, HDL, LDL, TC	9
Namvaran et al., 2011 [28]	Asian	101 (20.8)	51.44 (7.7)	27.33 (4.2)	Pioglitazone, 15	12	RT-PCR, TaqMan assay	HbA_1C_, FPG, TG, HDL, LDL, TC	8
Pei et al., 2013 [29]	Asian	67 (58.2)	56.63 (8.6)	25.15 (2.8)	Pioglitazone, 30	12	MALDI-TOF	HbA_1C_, FPG, TG, HDL, LDL, TC	8
Priya et al., 2016 [30]	Asian	30 (46.0)	53.2 (11.0)	26.25 (3.7)	Pioglitazone, 30	12	PCR	HbA_1C_, FPG	8

FPG: fasting plasma glucose; HbA_1C_: hemoglobin A_1C_; HDL: high-density lipoprotein cholesterol; LDL: low-density lipoprotein cholesterol; MALDI-TOF: matrix-assisted laser desorption/ionization time-of-flight; NOS: Newcastle–Ottawa score; PCR-RFLP: polymerase chain reaction–restriction fragment length polymorphism; RT-PCR: real time polymerase chain reaction; SD: standard deviation; TC: total cholesterol; TG: triglyceride.

## Data Availability

Not applicable.

## Supplementary Materials

The following supporting information can be downloaded at: https://www.mdpi.com/article/10.3390/pharmaceutics15061778/s1, Supplementary Table S1. Search strategy; Supplementary Table S2. Begg’s test and Egger’s test; Supplementary Table S3. Sensitivity analysis on the FPG outcome; Supplementary Table S4. Sensitivity analysis on the TC outcome; Supplementary Table S5. Sensitivity analysis on the HbA1C outcome; Supplementary Table S6. Sensitivity analysis on the TG outcome.
