# Supplementary material for: Correlation between PPARG Pro12Ala Polymorphism and Therapeutic Responses to Thiazolidinediones in Patients with Type 2 Diabetes: A Meta-Analysis
Source: Pharmaceutics. 2023 Jun 20;15(6):1778. doi: 10.3390/pharmaceutics15061778 (PMC10303709; doi:10.3390/pharmaceutics15061778)
Supplement: Supplementary file 1 [file pharmaceutics-15-01778-s001.zip › pharmaceutics-2413149-supplementary Final.pdf]

pharmaceutics

MDPI

Supplementary materials

Correlation between *PPARG* Pro12Ala polymorphism and therapeutic responses to thiazolidinediones in patients with type 2 diabetes: a meta-analysis

Eun Jeong Jang[1†], Da Hoon Lee[1†], Sae-Seul Im[2], Jeong Yee[1] and Hye Sun Gwak[1*]

Table S1. Search strategy.

No	Search term	Pubmed	Web of Science	Embase
#1	thiazolidinedion* OR (thiazolidine dion*) OR TZD OR glitazon* OR pioglitazon* OR rosiglitazon* OR troglitazon* OR lobeglitazon*	18,638	23,748	48,392
#2	polymorph* OR variant* OR mutation* OR genotyp* OR haplotyp* OR allele* OR SNP* OR pharmacogen* OR Pro12Ala OR rs1801282	1,799,285	1,847,836	2,359,231
#3	(peroxisome proliferator activated receptor gamma) OR (peroxisome proliferator activated receptor γ) OR (PPAR gamma) OR (PPARgamma) OR (PPARγ) OR (PPAR γ) OR PPARG	32,887	39,501	55,511
#4	#1 and #2	3,513	4,159	7,221
#5	glycemic OR glycaemic OR glucos*	726,068	612,884	1,082,599
#6	lipid* OR cholesterol* OR lipoprotein*	953,031	782,880	1,268,672
#7	(glycated hemoglobin) OR (glycated haemoglobin) OR (glycosylated hemoglobin) OR (glycosylated haemoglobin) OR (hemoglobin A1c) OR (haemoglobin A1c) OR HbA1c OR A1c OR glycohemoglobin OR glycohaemoglobin	77,204	56,209	161,732
#8	(fasting plasma glucos*) OR FPG OR (fasting blood glucos*) OR FBG OR (fasting blood sugar)	75,291	63,067	110,732
#9	(low density lipoprotein cholesterol*) OR (LDL cholesterol*) OR (LDL C) OR (low density lipoprotein*) OR LDL OR (beta lipoprotein cholesterol*)	143,810	129,409	263,387
#10	(high density lipoprotein cholesterol*) OR (HDL cholesterol*) OR (HDL C) OR (high density lipoprotein*) OR HDL OR (alpha lipoprotein cholesterol*)	121,431	110,124	233,036
#11	triglycerid* OR triacylglycerol* OR TG	210,829	294,575	369,807
#12	(total cholesterol*) OR TC	189,366	298,500	283,351
#13	#5 OR #6 OR #7 OR #8 OR #9 OR #10 OR #11 OR #12	1,734,675	1,723,340	2,401,785
#14	#1 AND #4 AND #13	201	293	509

Table S2. Begg's test and Egger's test.

Outcomes	P-values of Begg's test	P-values of Egger's test
HbA_{1C}	0.85	0.71
FPG	0.57	0.74
TG	0.62	0.90
HDL	1	0.96
LDL	0.62	0.51
TC	0.62	0.19

HbA_{1C}: hemoglobin A_{1C}, FPG: fasting plasma glucose, TG: triglycerides, LDL: low-density lipoprotein cholesterol, HDL: high-density lipoprotein cholesterol, TC: total cholesterol.

Table S3. Sensitivity analysis on the FPG outcome.

Excluded study	Heterogeneity I^2 (%)	Statistical model	Mean difference [95% CI]
None	59	Random	−10.91 [−19.82, −2.01]
Bluher et al. 2003	8	Fixed	−14.37 [−20.67, −8.07]
Hsieh et al. 2010	67	Random	−11.79 [−22.49, −1.10]
Kang et al. 2005	42	Fixed	−7.07 [−12.63, −1.52]
Namvaran et al. 2011	67	Random	−11.17 [−20.70, −1.65]
Pei et al. 2013	59	Random	−9.20 [−19.32, 0.92]
Priya et al. 2016	67	Random	−11.40 [−23.33, 0.53]

CI: confidence interval; FPG: fasting plasma glucose.

Table S4. Sensitivity analysis on the TC outcome.

Excluded study	Heterogeneity I^2 (%)	Statistical model	Mean difference [95% CI]
None	0	Fixed	6.40 [−0.05, 12.84]
Bluher et al. 2003	0	Fixed	5.48 [−1.51, 12.47]
Hsieh et al. 2010	0	Fixed	4.91 [−2.04, 11.86]
Kang et al. 2005	0	Fixed	12.43 [1.97, 22.88]
Namvaran et al. 2011	0	Fixed	6.31 [−0.29, 12.91]
Pei et al. 2013	0	Fixed	6.24 [−0.35, 12.82]

CI: confidence interval; TC: total cholesterol.

Table S5. Sensitivity analysis on the HbA_{1C} outcome.

Excluded study	Heterogeneity I^2 (%)	Statistical model	Mean difference [95% CI]
None	7	Fixed	−0.30 [−0.55, −0.05]
Bluher et al. 2003	0	Fixed	−0.39 [−0.66, −0.11]
Hsieh et al. 2010	23	Fixed	−0.34 [−0.65, −0.03]
Kang et al. 2005	0	Fixed	−0.23 [−0.50, 0.03]
Namvaran et al. 2011	19	Fixed	−0.31 [−0.57, −0.06]
Pei et al. 2013	11	Fixed	−0.26 [−0.52, 0.01]
Priya et al. 2016	23	Fixed	−0.40 [−0.92, 0.12]

CI: confidence interval; HbA_{1C}: hemoglobin A_{1C}.

Table S6. Sensitivity analysis on the TG outcome.

Excluded study	Heterogeneity I^2 (%)	Statistical model	Mean difference [95% CI]
None	6	Fixed	−26.88 [−41.30, −12.46]
Bluher et al. 2003	27	Fixed	−30.08 [−51.44, −8.73]
Hsieh et al. 2010	0	Fixed	−30.28 [−45.44, −15.12]
Kang et al. 2005	29	Fixed	−27.30 [−42.18, −12.43]
Namvaran et al. 2011	29	Fixed	−26.25 [−41.82, −10.67]
Pei et al. 2013	0	Fixed	−21.69 [−37.39, −5.99]

CI: confidence interval; TG: triglyceride.

Table S7. Sensitivity analysis on the HDL outcome.

Excluded study	Heterogeneity I^2 (%)	Statistical model	Mean difference [95% CI]
None	0	Fixed	0.31 [−1.62, 2.23]
Bluher et al. 2003	0	Fixed	0.94 [−1.84, 3.73]
Hsieh et al. 2010	0	Fixed	−0.14 [−2.37, 2.09]
Kang et al. 2005	0	Fixed	0.18 [−1.88, 2.24]
Namvaran et al. 2011	0	Fixed	0.46 [−1.55, 2.46]
Pei et al. 2013	0	Fixed	0.31 [−1.62, 2.25]

CI: confidence interval; HDL: high-density lipoprotein cholesterol.

Table S8. Sensitivity analysis on the LDL outcome.

Excluded study	Heterogeneity I^2 (%)	Statistical model	Mean difference [95% CI]
None	0	Fixed	6.69 [−0.90 14.29]
Bluher et al. 2003	0	Fixed	6.60 [−1.82, 15.04]
Hsieh et al. 2010	0	Fixed	6.48 [−2.05, 15.02]
Kang et al. 2005	0	Fixed	6.94 [−2.34, 16.22]
Namvaran et al. 2011	0	Fixed	6.82 [−1.69, 15.32]
Pei et al. 2013	0	Fixed	6.68 [−1.18, 14.54]

CI: confidence interval; LDL: low-density lipoprotein cholesterol.
